# Effects of Photochromic Furan-Based Diarylethenes on Gold Nanoparticles Aggregation

**DOI:** 10.1186/s11671-017-2044-6

**Published:** 2017-04-13

**Authors:** Alina Khodko, Nataliya Kachalova, Sergiy Scherbakov, Anna Eremenko, Iuliia Mukha

**Affiliations:** 1 Institute of Physics of National Academy of Sciences of Ukraine, prospect Nauky, 46, Kyiv, 03028 Ukraine; 2 M.G. Kholodny Institute of Botany of National Academy of Sciences of Ukraine, Tereshchenkivska st., 2, Kyiv, 01601 Ukraine; 3 Chuiko Institute of Surface Chemistry of National Academy of Sciences of Ukraine, General Naumov Str., 17, Kyiv, 03164 Ukraine

**Keywords:** Photochromic Molecule, Diarylethenes/Difurylethenes, Gold Nanoparticles, Surface Plasmon Resonance, UV/VIS Absorption Spectroscopy, Transmission Electron Microscopy, 81.07.Pr (Organic-inorganic hybrid nanostructures), 78.67.Bf (Nanocrystalsnanoparticles, and nanoclusters), 78.67.-n (Optical properties of low-dimensional, mesoscopic, and nanoscale materials and structures)

## Abstract

The photochromic properties of furan-based diarylethenes and their interaction with citrate-capped gold nanoparticles were investigated by ultraviolet/visible absorption spectroscopy and transmission electron microscopy. We identified the optimal concentration of diarylethenes in water-ethanol mixture required for stability of colloidal systems. Nanoparticles coupled with diarylethene derivatives exhibit a new surface plasmon resonance band coming from their aggregation. We analyzed the effects of functional side-chain groups on aggregation process. These results can be considered as a basis for further designing of novel hybrid nanomaterials and optoelectronic elements.

**Electronic supplementary material:**

The online version of this article (doi:10.1186/s11671-017-2044-6) contains supplementary material, which is available to authorized users.

## Background

**Fig. 1:**
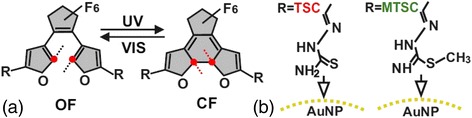
**a** The schematic representation of photoinduced switching between open-ring (OF) and closed-ring (CF) forms of DAEs under ultraviolet (UV) and visible (VIS) light illumination. **b** The structures of TSC (*left*) and MTSC (*right*) side-chain groups

The diarylethene derivatives (DAEs)—photochromic molecules [1, 2] that can be reversibly switched between open-ring (OF) and closed-ring (CF) forms by external optical and/or electrical stimulation (Fig. 1a), attract considerable attention since these molecular switches grafted on metal nanoparticles [3] are promising base for optoelectronic elements [4, 5], smart materials [6, 7], and molecular machines [8, 9]. The gold nanoparticles connected through the photochromic molecules can serve as a conducting path between electrodes [10, 11]. But, the localized surface plasmon resonance can inhibit the switching properties through the molecule-metal interactions [12]. The effects, which underlie these interactions, are critique for further applications and require the complementary investigation.

In this work, we studied the interaction of photochromic furan-based diarylethenes (DAEs) with citrate-capped gold nanoparticles (Au NPs). Recently, their conductance on a single-molecule level was investigated by break-junction experiments at low temperatures [10]. DAEs contained thiosemicarbazone (TSC) and methylthiosemicarbazone (MTSC) side-chain groups (Fig. 1b) that were used to extend π-system of photochromic switches [13, 14] and to decouple the molecular switch from the gold electrodes and, thus, to avoid the unintended quenching of the excited state [11, 15]. Organic-inorganic hybrid nanostructures obtained in solution can be easily characterized and deposited on tailored surface in a controllable way. Thus, we chose the colloid state in water-ethanol mixture to investigate the DAE/Au NPs systems.

## Methods

The furan-based DAEs—1,2-bis[2-methyl-5-[(*E*)-(2-thiocarbamoylhydrazono)methyl]-furan-3-yl]-perfluorocyclopentene (C5F-TSC) and 1,2-bis[2-methyl-5-[(*E*)-(1-methylthio-1-iminomethylhydrazono)methyl]-furan-3-yl]-perfluorocyclopentene (C5F-MTSC), were synthesized and purified according to [13]. The CF and OF for both molecules are thermally stable and reversibly switchable and have appropriate photoconversion quantum yields [15] which make these molecules suitable for further implementation in the molecular-scaled flexible devices with optically modulated properties [5].

The colloidal solutions of gold nanoparticles were synthesized via chemical reduction of tetrachlorauric acid with trisodium citrate according to the Turkevich method [16, 17]. Synthesis of 20 mL of Au NPs was carried in aqueous solutions with concentrations of gold in the final colloid of *C*_Au_ = 2.5 × 10^−4^ M when stirred and heated to boiling. The obtained colloidal solution of gold nanoparticles was cooled to room temperature for 5 min after the immersion of 1 mL of citrate with *C*_citrate_ = 8.5 × 10^−4^ M.

The ethanol solutions of C5F-TSC and C5F-MTSC with concentration *C* = 10^−5^ M were used to investigate the photochemical properties of both molecules by ultraviolet/visible (UV/VIS) absorption spectroscopy. To initiate the ring-closing reaction, the mercury lamp (PRK-4) with the appropriate combination of filters was used as UV light source (*λ* = 365 nm), while the LED source (*λ* = 430–750 nm) was used to start the reverse ring-opening reaction. All solutions were stored in the dark to avoid uncontrolled photochromic reactions, which may occur under the influence of natural or/and artificial light.

The water-ethanol solutions of molecules were prepared by mixing the ethanol solutions of CF or OF of C5F-TSC and C5F-MTSC with distilled water (i.e., the volume ratio of water to ethanol was equal to 4:1). The same procedure was performed to investigate the interaction of Au NPs with DAEs: gold colloid was mixed with previously irradiated ethanol solutions of C5F-TSC and C5F-MTSC; the quantity of ethanol was no more than 20% vol. Namely, the volume ratios of water to ethanol were equal to 4:1 and 5:1 for two series. The two sets of samples were prepared in the same experimental conditions. The DAE’s concentration was the only one parameter that was varied in a range from *C*_DAE_ = 10^−7^ to 10^−5^ M, while the concentration of gold *C*_Au_ = 2 × 10^−4^ M was constant for all series.

The photoinduced transformation of molecules and their interaction with Au NPs were measured by the Lambda 35 UV/VIS spectrometer (Perkin-Elmer Instruments, USA) in 1-cm quartz cells. The size and morphology of nanoparticles and their aggregates were characterized by the transmission electron microscope (TEM) JEM-1230 (JEOL, Japan) with an accelerating voltage of 80 kV. One microliter of colloid was placed on carbon-coated copper grids and dried at room temperature. The program ImageJ was used to calculate the distribution of particles.

## Results and Discussion

### Solvent Effects on Photochromic Properties of Diarylethene Derivatives

**Fig. 2:**
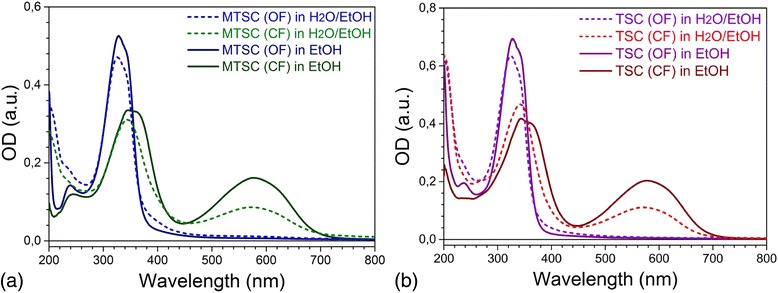
The photoinduced transformations between OF and CF represented in absorbance spectra of **a** C5F-MTSC and **b** C5F-TSC solved in ethanol and water-ethanol solutions, *C*_DAE_ = 10^−5^ M

The adsorption spectra of both C5F-TSC and C5F-MTSC in CF show the broad absorption band located in the visible range. They appear after UV light illumination of the colorless solutions of OF and indicate the ring-closing reaction of photochromic core (Fig. 2). Although studied DAEs have the same photochromic core and differ only with one methyl group (C5F-MTSC), bonded to sulfur atom in molecular linkers (Fig. 1b), they show the different optical response.

The shape of absorption bands is similar, but the molar absorptivity in case of TSC substituent is almost 1.5 times higher compare to MTSC. The absorbance maximum of C5F-TSC in a visible range is located at *λ*_max_ = 580 nm in ethanol, while of C5F-MTSC is slightly blue shifted to *λ*_max_ = 575 nm. This shift of Δ*λ* = 5 nm is also observed for more polar water-ethanol mixture; the maxima are located at *λ*_max_ = 575 and *λ*_max_ = 570 nm, correspondingly.

We found that the photoinduced ring-closing reaction of studied DAEs also occurs in water-ethanol mixture. The cyclization process proceeds two times more effective in ethanol compared to more polar water-ethanol mixture for both DAEs. It happens since in less polar solvents the ring-closure reaction proceeds easier, since the exited state of OF is stabilized in planar conformation close to the structure of CF, opposed to twisted form in polar solvents [18, 19]. Considering that DAEs grafted on gold nanoparticles can absorb at the different orientation and adsorption sites [20, 21], twisted form of DAE existing in more polar solvent could be advantageous. Firstly, the less quantity of ethanol causes the minor NPs aggregation at aqueous gold colloid. Secondly, in case of more polar water-ethanol solvent, the linkers of TSC and MTSC are more deployed in the space presumably promoting bonding of the molecules with two different nanoparticles. Thus, for further experiments with Au NPs, water-ethanol mixture can be used as a solvent.

### DAE Interaction with Gold Nanoparticles

**Fig. 3:**
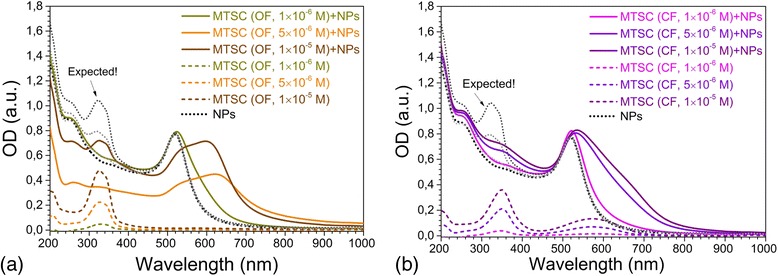
Changes in absorption spectra of gold nanoparticles influenced by C5F-MTSC in OF (**a**) and CF (**b**); *dotted lines* show the spectra of C5F-MTSC in control measurements. The volume ratio of water to ethanol was equal to 4:1. The DAE concentrations were *C*_DAE_ = 1 × 10^−6^, 5 × 10^−6^, 1 × 10^−5^ M. The concentration of gold *C*_Au_ = 2 × 10^−4^ M was constant for all series

The colloidal solutions of citrate-capped gold nanoparticles had inherent red color accompanied by the presence of localized surface plasmon resonance (LSPR) band [22, 23] in absorption spectrum with intensive maxima at *λ* = 519 nm (Fig. 3). The particles were mainly formed with the average size of 15–20 nm according to the TEM measurements. Stability of Au NPs was provided by the negative charge of citrate shell, which prevented them from aggregation in solutions.

The C5F-MTSC in OF (*C* = 5 × 10^−6^ M and *C* = 10^−5^ M) caused strong aggregation of nanoparticles. The second plasmon resonance band of gold appeared as a shoulder and then as maximum near *λ* = 600 nm. This long wavelength mode is the excitation of plasmon resonance along the linear chains of nanoparticles (chain mode) [24] that is intrinsic for gold aggregates with different cross-linking agents [25, 26]. In our case, it was due to the growth of aggregates of Au NPs caused by DAE binding (Fig. 3a). Changes in absorption spectra were accompanied by dramatic color change of solutions from red (citrate-capped Au NPs not functionalized with DAE) to deep violet (Additional file 1: Figure S1). The absorbance spectra of Au NPs/DAE system differed from the expected ones (Fig. 3 dotted lines) calculated as the sum of absorbance of isolated Au NPs and C5F-MTSC as non-interacting substances. Thus, we proved that the strong interaction between Au NPs and DAEs occurred. OF of DAEs with MTSC substituent interacted with Au NPs in nonlinear manner. Twice less amount of DAE, *C* = 5 × 10^−6^ M, caused total aggregation of NPs to dark blue precipitate. The second plasmon resonance band maximum of gold was shifted from 610 to 634 nm during 1 h followed by its total disappearance.

The C5F-MTSC in CF interacted with Au NPs in a completely different way. In the absorption spectra, we observed LSPR bands with a slight long wavelength shoulder, but with maxima located at *λ* = 532 nm (*C* = 5 × 10^−6^ M) and *λ* = 535 nm (*C* = 1 × 10^−5^ M) (Fig. 3b), we found that C5F-MTSC at concentration *C* = 10^−6^ M caused the formation of stable colloids in both OF and CF. Their maxima were located at *λ* = 522 nm for CF and *λ* = 524 nm for OF, close to the maximum of citrate-capped gold nanoparticles. With the aim to obtain the stable colloids of Au NPs functionalized with photochromic molecules for further experiments, we decreased the concentration of DAEs (up to *C* = 0.1 × 10^−6^ M) and used less volume of ethanol to avoid NPs aggregation (from 4:1 to 5:1 water to ethanol volumic ratio).

The first observation evidenced that DAE derivatives with TSC and MTSC side chains interacted with gold nanoparticles and in both cases the strong aggregation of nanoparticles occurred in case of OF. One more important finding is that the optimal concentration of DAE needed to form stable colloids was determined, *C*_DAE_ = 10^−6^ M.

**Fig. 4:**
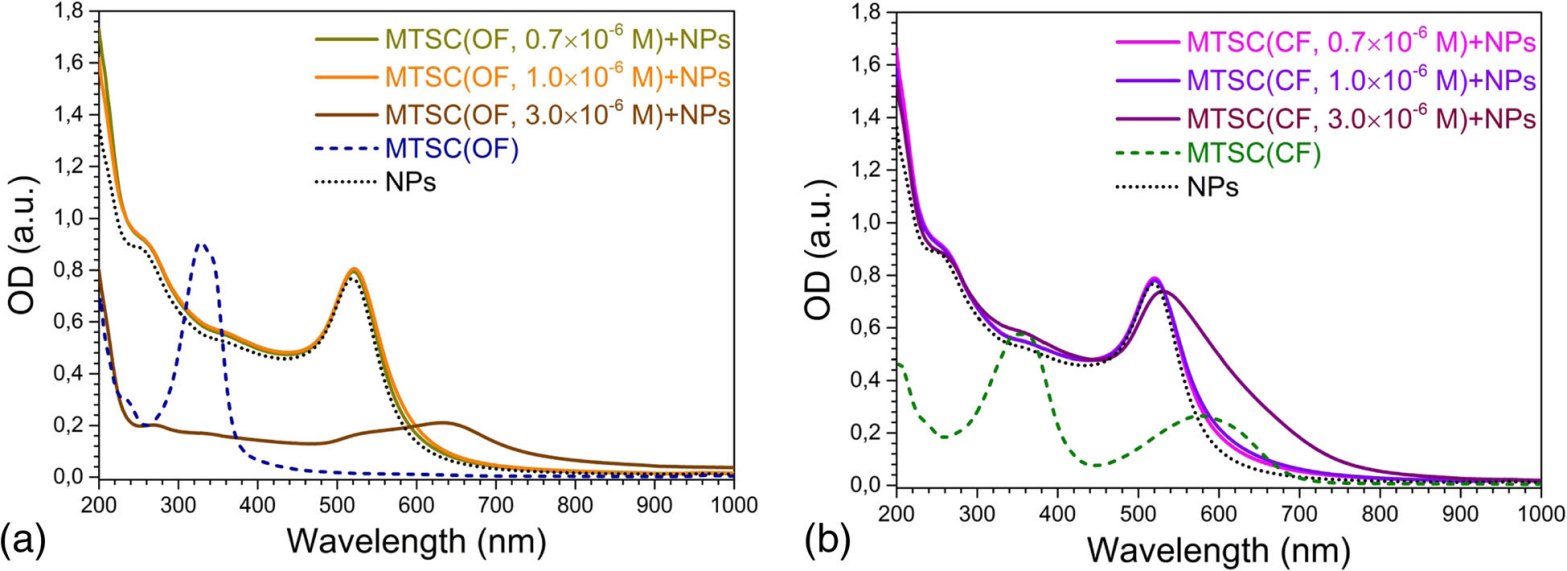
Changes in absorption spectra of gold nanoparticles influenced by C5F-MTSC in OF (**a**) and CF (**b**). The volume ratio of water to ethanol was equal to 5:1. The concentration of DAEs was *C*_DAE_ = 0.7 × 10^−6^, 1.0 × 10^−6^, 3.0 × 10^−6^ M, but the control spectra C5F-MTSC in OF and CF was measured at *C*_DAE_ = 10^−5^ M. The concentration of gold *C*_Au_ = 2 × 10^−4^ M was constant for all series

**Fig. 5:**
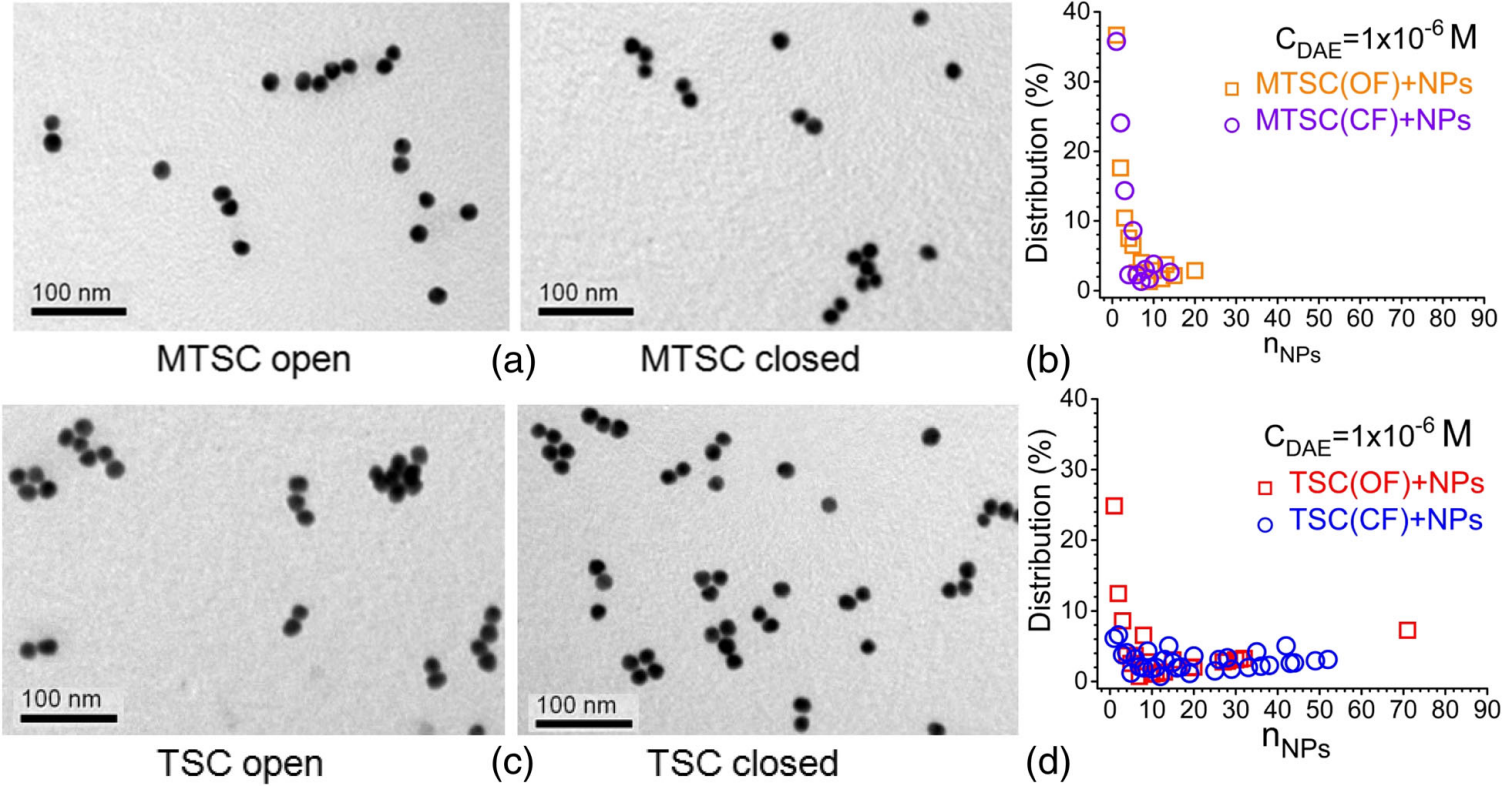
TEM images of obtained colloids, where C5F-MTSC (**a**) and C5F-TSC (**c**) interacted with Au NPs in OF and CF at *C*_DAE_ = 1 × 10^−6^ M. The distribution of aggregates by quantity of NPs per assembly for C5F-MTSC (**b**) and C5F-TSC (**d**)

No significant variations in absorption spectra of gold nanoparticles under the action of both OF and CF of C5F-MTSC were detected for DAEs concentration up to *C*_DAE_ = 1.0 × 10^−6^ (Fig. 4), and samples had the same red coloration (Additional file 2: Figure S2). The colloids containing C5F-MTSC in OF and CF with *C*_DAE_ = 1 × 10^−6^ M were investigated by TEM. We detected a large number of small aggregates up to 20 NPs in assembly for both samples (Fig. 5), and more than 35% of them were monomers (693 NPs were analyzed for the system with DAEs in OF and 523 NPs for the system with DAEs in CF).

**Fig. 6:**
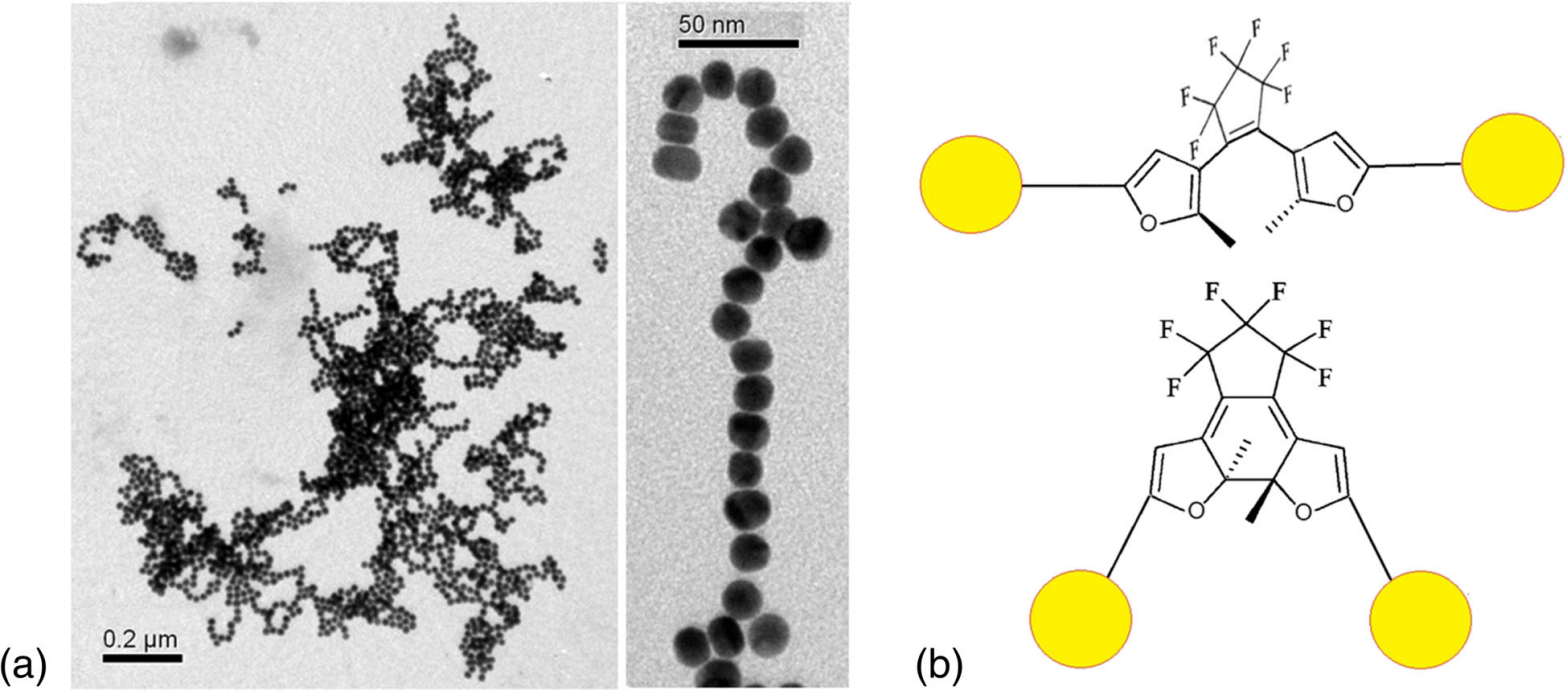
**a** TEM images of Au NPs/C5F-MTSC colloid, where DAE interacted in CF at *C*_DAE_ = 3 × 10^−6^ mol·L^−1^. **b** Model representation of interaction between DAEs (OF and CF) with Au NPs

But, at *C*_DAE_ = 3 × 10^−6^ M, the most crucial changes occurred (Fig. 4a, b). In the case of OF, LSPR band of gold at 634 nm disappeared in time. Au NPs were totally aggregated leaving blue-gray precipitate. It was not possible to analyze this sample by TEM. At the same time, CF promoted the formation of chain-like structures of nanoparticles and most of nanoparticles gathered in huge assemblies (Fig. 6) remaining stability of violet colloid with *λ*_max_ = 531 nm. Such dramatic and inconsistent change in stability possibly is connected with transformation of citrate-stabilizing shell and should be a subject of further investigation.

**Fig. 7:**
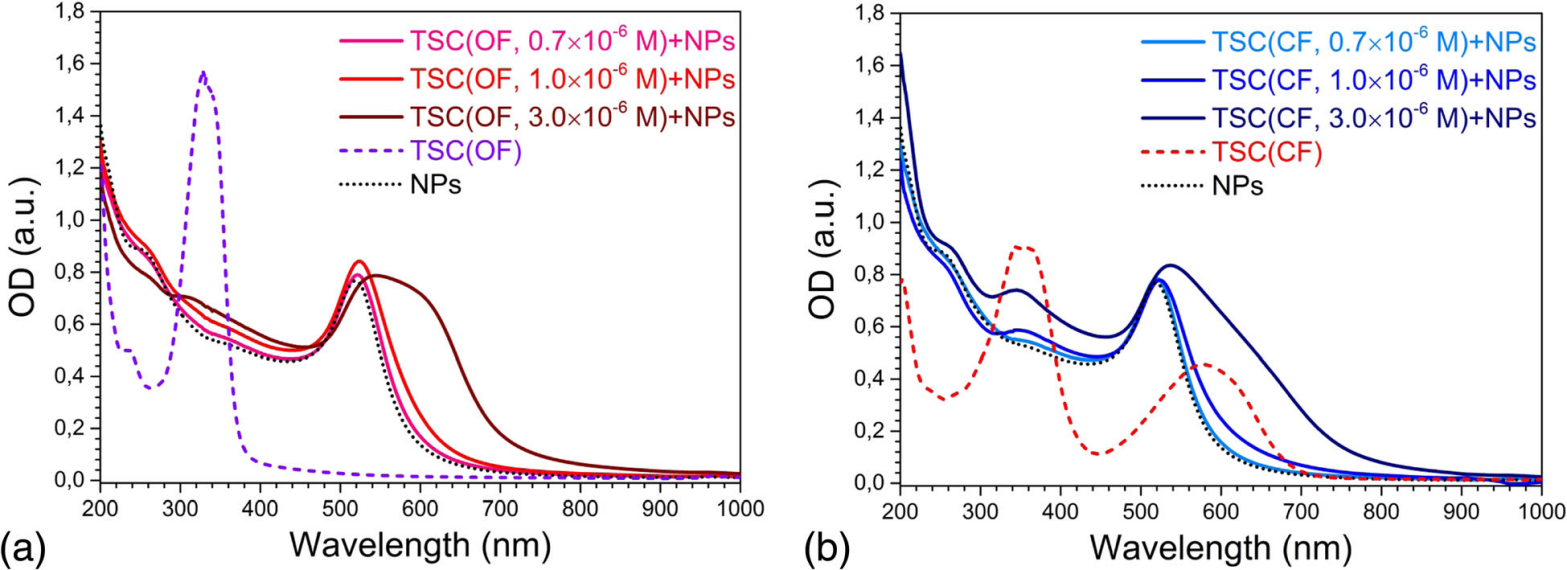
Changes in absorption spectra of gold nanoparticles influenced by C5F-TSC in OF (**a**) and CF (**b**); the volume ratio of water to ethanol was equal to 5:1. The concentration of DAEs were *C*_DAE_ = 0.7 × 10^−6^, 1.0 × 10^−6^, 3.0 × 10^−6^ M, but the control spectra C5F-TSC in OF and CF was measured at *C*_DAE_ = 10^−5^ M. The concentration of gold, *C*_Au_ = 2 × 10^−4^ M was constant for all series

The C5F-TSC, at *C*_DAE_ = 3 × 10^−6^ M also caused aggregation of particles, but not so strong compared to C5F-MTSC. The LSPR band maximum of Au NPs with OF of C5F-TSC was located at 546 nm having a big shoulder near 600 nm (Fig. 7a). Colloid of Au NPs with OF of C5F-TSC had violet coloration (Additional file 3: Figure S3) as well as one of Au NPs with CF. But for CF of C5F-TSC, LSPR band maximum was less shifted, *λ*_max_ = 537 nm, with a less prominent shoulder compared to OF (Fig. 7b).

**Fig. 8:**
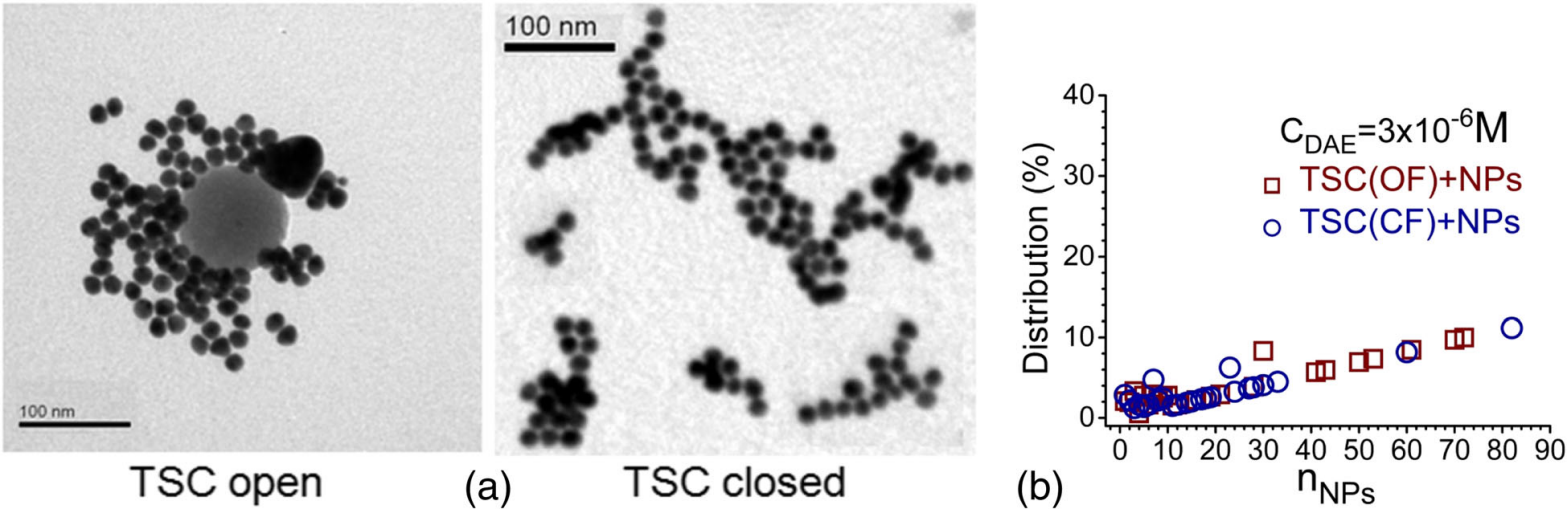
**a** TEM images of obtained colloids, where C5F-TSC interacted with Au NPs in OF and CF at *C*_DAE_ = 3 × 10^-6^ M. **b** The distribution of aggregates by quantity of NPs per assembly

According to the TEM measurements, more than half of the particles (53%) in violet colloid with C5F-TSC at *C*_DAE_ = 3 × 10^−6^ M in OF were organized in assemblies with *n* > 40 NPs. At the same time, C5F-TSC in CF formed near 20% of such assemblies (Fig. 8). The red colloids at *C*_DAE_ = 1 × 10^−6^ M with both OF and CF of C5F-TSC contained assemblies mainly up to 50 NPs at almost the same amount (721/978 NPs for OF and 735/1666 NPs for CF were analyzed) (Fig. 5c, d).

Thus, there is a clear difference in the behavior of two DAEs caused by the structure of their side-chain groups as well as their conformational forms.

The OF is expected to be more spatially distributed due to the flexibility of side-chain groups. The spatial structures of studied DAE obtained with ChemDraw MM2 energy minimization are shown in Additional file 4: Figure S4.

We can see that molecular linkers are more deployed in the space (placed from “different sides” relative to the core) in case of OF for both molecules. Moreover, the polar solvent causes the stabilization of the molecule in twisted conformation. Hence, the possibility of interaction of one DAE molecule with two different gold nanoparticles is higher compared to its CF. Therefore, OF can cause stronger aggregation that we observed in absorbance spectra and TEM images.

Comparing TSC and MTSC side-chain groups, their donating ability should be taken into account. The number of bonding modes has been observed for the thiosemicarbazones in their neutral or anionic forms [27]. Thiosemicarbazone moiety have mainly bidentate nature. Adsorption of TSC group on the surface of metal can occur through sulfur and nitrogen atoms. In its turn, MTSC group is bound to bind with gold through nitrogen(s) while sulfur is not involved, as was shown also for complexes of *S*-methylthiosemicarbazone derivatives with other metals [28–30].

The electron-donating ability of a molecule is often associated with the energy of high occupied molecular orbital (HOMO), and less negative HOMO energy and smaller energy gap (E_HOMO_-E_LUMO_) are expected to appear as stronger chemisorption [31]. ChemDraw calculations allow us to estimate the tendency and difference between two studied molecules. Although, to obtain the more accurate matches with experimental data, further calculations are required. According to the calculated HOMO-LUMO values, energy gaps of both OF and CF of C5F-MTSC are bigger than those for C5F-TSC. It correlates with positions of DAE maxima in absorption spectra (Fig. 2): C5F-MTSC is blue-shifted compared to C5F-TSC indicating that more energy is needed to reach the exited state. Also, the HOMO energy levels of OF and CF are higher for thiosemicarbazone derivative confirming its stronger donating properties.

Thus diarylethene derivatives with TSC and MTSC side-chain groups despite their similar structure have different optical response and interact variously while binding to gold nanoparticles.

## Conclusions

The water-ethanol mixture can be used as a solvent for investigation of effect of DAE on Au NPs and is advantageous due to more twisted orientation of photochromic molecules in more polar solvent. The interaction of DAE derivatives with TSC and MTSC side chains with citrate-capped gold nanoparticles in OF and CF has nonlinear manner. Both molecules cause aggregation of nanoparticles stronger in case of OF at the concentration of *C*_DAE_ = 3 × 10^−6^ mol·L^−1^ that is associated with spatial distribution of molecular linkers. At the same time, the colloids with lower concentration of C5F-MTSC and C5F-TSC, *C*_DAE_ = 1 × 10^−6^ mol·L^−1^, maintain stability more than half a year. Larger aggregates are formed in case of TSC side chains (up to 80 NPs per assembly) compared to MTSC tail (up to 20 NPs per assembly) due to the higher donating nature of the first one.

## Additional Files

Additional file 1: Figure S1. The color changes that represents the interection of DAEs with AuNPs: (a) C5F-MTSC and (b) C5F-TSC. The volume ratio water to ethanol is equal to 4:1, *C*_DAE_ = 1 × 10^−6^, 5 × 10^−6^, 1 × 10^−5^ mol·L^−1^. The concentration of gold *C*_Au_ = 2 × 10^−4^ mol·L^−1^ was constant for all series. The spectra of the corresponding solutions are presented in Fig. 3. (PPT 128 kb)

Additional file 2: Figure S2. Changes in color of the solutions, where gold nanoparticles were influenced by C5F-MTSC in open-ring (a) and closed-ring (b) forms. The volume ratio of water to ethanol = 5:1, *C*_DAE_ = 0.7 × 10^−6^, 1.0 × 10^−6^, 3.0 × 10^−6^ mol·L^−1^. The concentration of gold *C*_Au_ = 2 × 10^−4^ mol·L^−1^ was constant for all series. The spectra of the corresponding solutions are presented in Fig. 4. (PPT 202 kb)

Additional file 3: Figure S3. Changes in color of the solutions, where gold nanoparticles were influenced by C5F-TSC in open-ring (a) and closed-ring (b) forms. The volume ratio of water to ethanol = 5:1, *C*_DAE_ = 0.7 × 10^−6^, 1.0 × 10^−6^, 3.0 × 10^−6^ mol·L^−1^. The concentration of gold C_Au_ = 2 × 10^−4^ mol·L^−1^ was constant for all series. The spectra of the corresponding solutions are presented in Fig. 7. (PPT 214 kb)

Additional file 4: Figure S4. The spatial structures of C5F-MTSC (top) and C5F-TSC (bottom) in open-ring (left) and closed-ring (right) states obtained with ChemDraw MM2 energy minimization. (PPT 219 kb)

## Abbreviations

Au NPs: Gold nanoparticles
C5F-MTSC: Furan-based diarylethene with methylthiosemicarbazone side chains
C5F-TSC: Furan-based diarylethene with thiosemicarbazone side chains
CF: Closed-ring form
DAEs: Diarylethenes
HOMO: High occupied molecular orbital
LSPR: Localized surface plasmon resonance
LUMO: Lowest unoccupied molecular orbital
MTSC: Methylthiosemicarbazone
OF: Open-ring form
TEM: Transmission electron microscope
TSC: Thiosemicarbazone
UV/VIS: Ultraviolet/visible
