# Supplementary figures and images for: Effects of Photochromic Furan-Based Diarylethenes on Gold Nanoparticles Aggregation
Source: Nanoscale Res Lett. 2017 Apr 13;12:271. doi: 10.1186/s11671-017-2044-6 (PMC5391340; doi:10.1186/s11671-017-2044-6)

## Slide 1
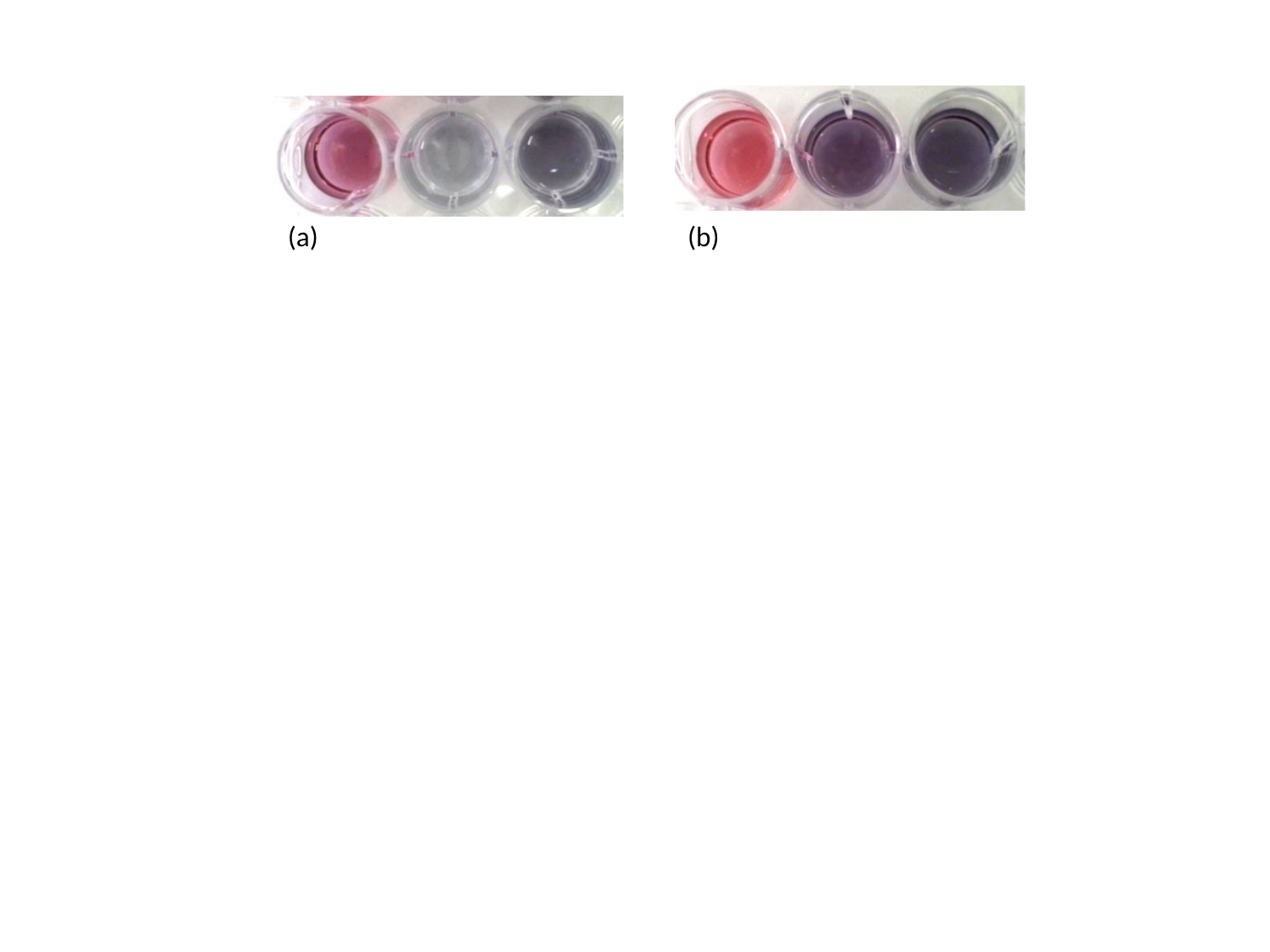

(a)
(b)

Supplement: Supplementary file 1 — The color changes that represents the interection of DAEs with AuNPs: (a) C5F-MTSC and (b) C5F-TSC. The volume ratio water to ethanol is equal to 4:1, C DAE = 1 × 10^−6^, 5 × 10^−6^, 1 × 10^−5^ mol·L−1. The concentration of gold C Au = 2 × 10^−4^ mol·L−1 was constant for all series. The spectra of the corresponding solutions are presented in Fig. 3. (PPT 128 kb) [file 11671_2017_2044_MOESM1_ESM.ppt]

## Slide 1
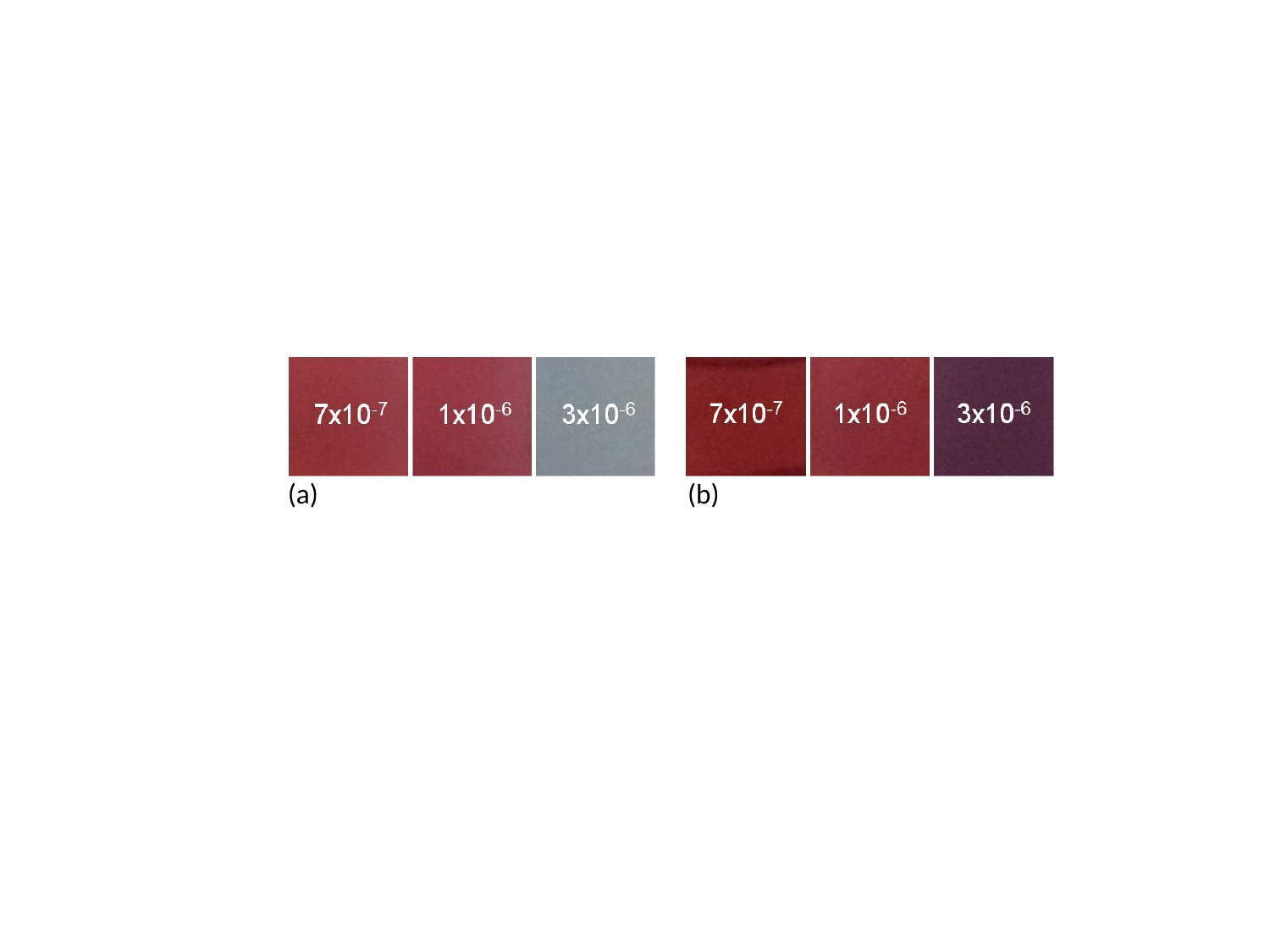

(a)
(b)

Supplement: Supplementary file 2 — Changes in color of the solutions, where gold nanoparticles were influenced by C5F-MTSC in open-ring (a) and closed-ring (b) forms. The volume ratio of water to ethanol = 5:1, C DAE = 0.7 × 10^−6^, 1.0 × 10^−6^, 3.0 × 10^−6^ mol·L−1. The concentration of gold C Au = 2 × 10^−4^ mol·L−1 was constant for all series. The spectra of the corresponding solutions are presented in Fig. 4. (PPT 202 kb) [file 11671_2017_2044_MOESM2_ESM.ppt]

## Slide 1
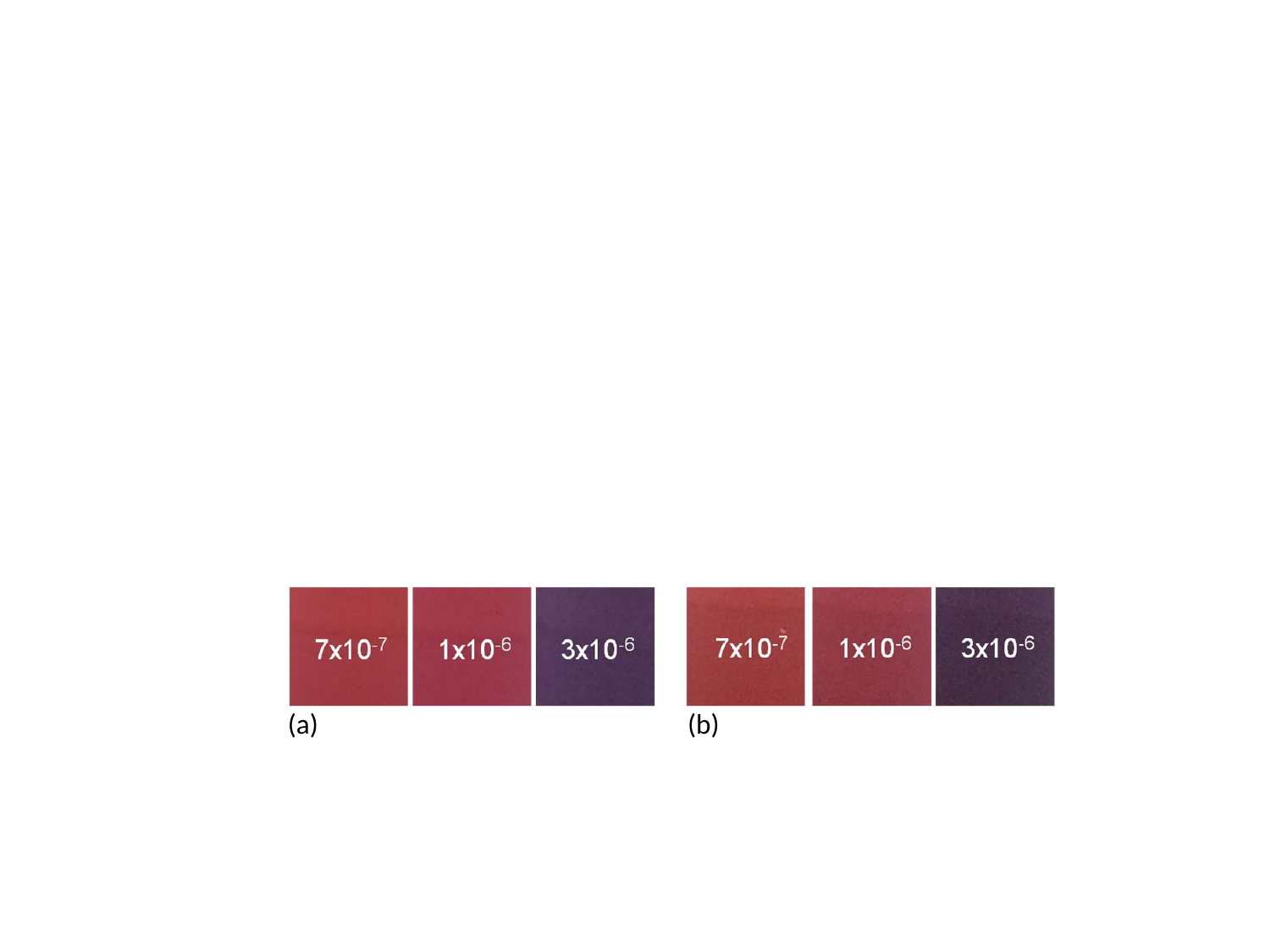

(a)
(b)

Supplement: Supplementary file 3 — Changes in color of the solutions, where gold nanoparticles were influenced by C5F-TSC in open-ring (a) and closed-ring (b) forms. The volume ratio of water to ethanol = 5:1, C DAE = 0.7 × 10^−6^, 1.0 × 10^−6^, 3.0 × 10^−6^ mol·L−1. The concentration of gold CAu = 2 × 10^−4^ mol·L−1 was constant for all series. The spectra of the corresponding solutions are presented in Fig. 7. (PPT 214 kb) [file 11671_2017_2044_MOESM3_ESM.ppt]

## Slide 1
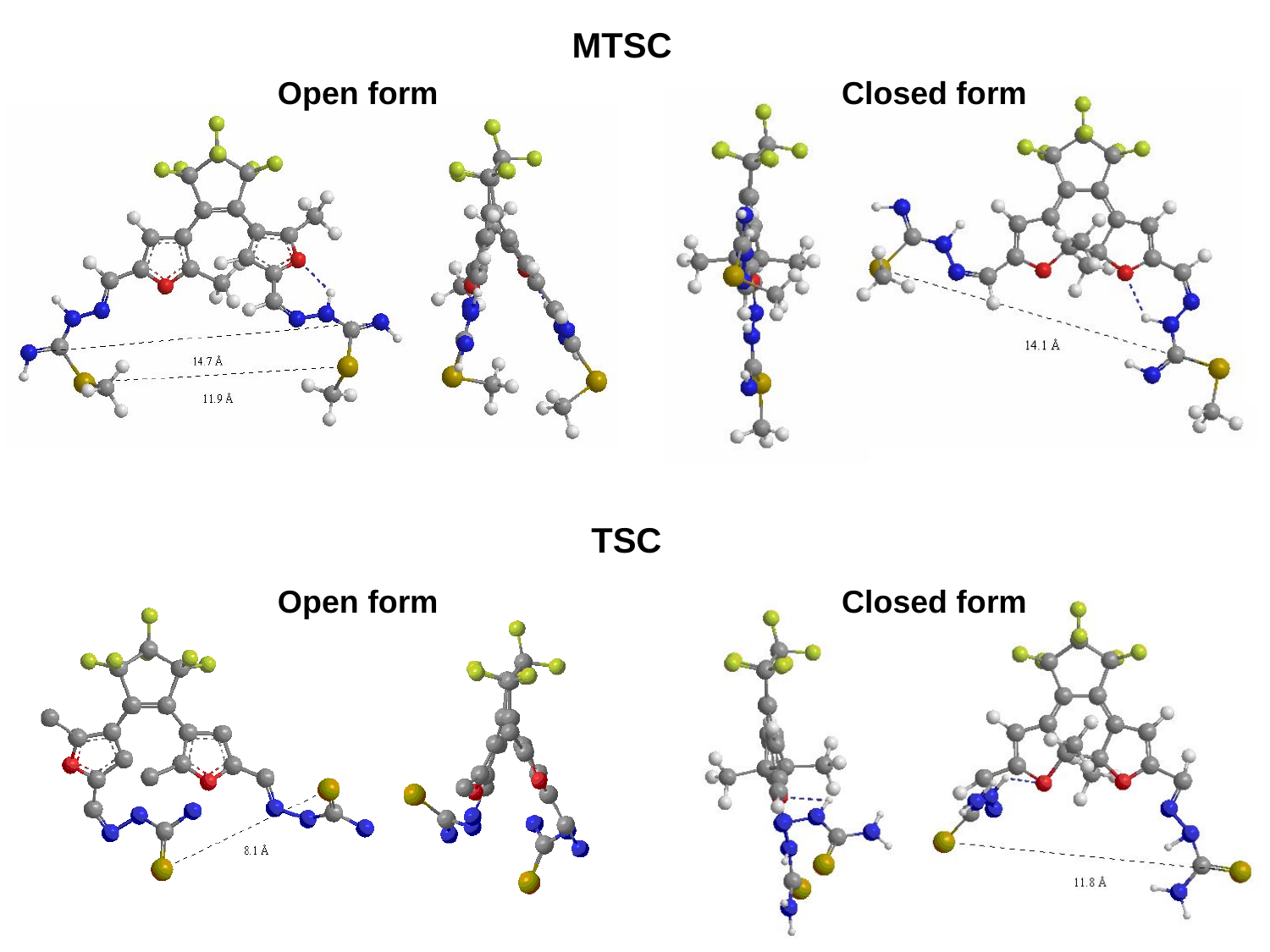

MTSC
Open form
Closed form
TSC
Open form
Closed form

Supplement: Supplementary file 4 — The spatial structures of C5F-MTSC (top) and C5F-TSC (bottom) in open-ring (left) and closed-ring (right) states obtained with ChemDraw MM2 energy minimization. (PPT 219 kb) [file 11671_2017_2044_MOESM4_ESM.ppt]
